# Le genre *Tunga* Jarocki, 1838 (Siphonaptera : Tungidae). I – Taxonomie, phylogénie, écologie, rôle pathogène

**DOI:** 10.1051/parasite/2012194297

**Published:** 2012-11-15

**Authors:** J.-C. Beaucournu, B. Degeilh, T. Mergey, S. Muñoz-Leal, D. González-Acuña

**Affiliations:** 1 Laboratoire de Parasitologie et Zoologie appliquée, Faculté de Médecine et Institut de Parasitologie de l’Ouest 2, avenue du Professeur Léon Bernard 35043 Rennes Cedex France; 2 Laboratoire de Parasitologie, Mycologie et Immunologie Parasitaire, CHRU 2, rue Henri Le Guilloux 35033 Rennes Cedex France; 3 Facultad de Ciencias Veterinarias, Universidad de Concepción casilla 537 Chillán Chili

**Keywords:** *Tunga*, Siphonaptera, taxonomie, écologie, phylogénie, rôle pathogène, *Tunga*, Siphonaptera, taxonomy, ecology, phylogeny, pathogenicity

## Abstract

Pour la première fois, les 12 espèces actuellement décrites dans le genre *Tunga* sont étudiées sur le plan de la taxonomie et de la répartition. Divers aspects de leur biologie et leur rôle pathogène sont également envisagés, et en particulier leur phylogénie, leur chorologie, leur phénologie, leur sexe-ratio et leurs *dermecos*.

## Abstract (en)

This is the first review of the taxonomy and geographical range of the 12 known species of the genus *Tunga*. Their biology and pathogenic roles are considered, with particular emphasis on their phylogeny, chorology, phenology, sex-ratio, and dermecos.

## Introduction

“*Chique, s. f., ciron qui entre dans la chair, et y cause des démangeaisons insupportables ; tabac à mâcher.”*

“*Ciron, s. m., très petit insecte.*”

(Sauger-Préneuf et Détournel, *Vocabulaire français*, 1839)

Linné dans le *Systema naturae* de 1758 nomme deux espèces de puces. La première est *Pulex irritans*, l’un de nos “*Compagnons de toujours*” selon l’expression de Doby, 1996, la seconde est *Pulex penetrans*, la chique, cette puce originale qui s’enfouit dans son hôte au lieu de se promener à la surface de son corps, ou dans son lit ! Toutefois, des différences morphologiques et biologiques manifestes amenèrent Jarocki, en 1838, à en faire le type d’un genre nouveau, *Tunga*. En utilisant ce nom, Jarocki ne faisait d’ailleurs que s’inspirer des données recueillies par Linné : “*marcgr* & *piso, bras., Tunga*”, ce que l’on peut approximativement traduire par : “*la puce dont* *Marcgraf (1648)* *et* *Pison (1658)* *disent, qu’au Brésil, le nom est Tunga*”.

Ce fut Enderlein qui décrivit, en, 1901, la deuxième espèce du genre, *T. caecata*, ainsi nommée car cette espèce possède un oeil petit et sans pigment, à l’inverse de ce que l’on observe chez *T. penetrans*. Depuis, dix autres taxons ont été découverts (un 13^ème^ est en cours de description), l’immense majorité en région néotropicale, mais une le fut dans le sud de la région néarctique et deux en région paléarctique orientale.

Quel que soit le rang taxonomique que l’on accorde au genre *Tunga*, sous-Famille, Famille, super-Famille (Lewis, 1998, 2009) ou “groupe-frère” des Siphonaptères (Whiting et al., 2008), cela ne clarifie guère sa position. De même, que l’on place, ou non, à son côté *Hectopsylla* Frauenfeld, 1860, voire même *Neotunga* Smit, 1962c et/ou *Phacopsylla* Beaucournu & Horak, 1994 (Lewis, *opp. cit.*) ne facilite en rien, à notre avis, l’approche de ce genre. À l’exception de *Neotunga* Smit (1962), puce afrotropicale chez qui, malheureusement, seule la femelle est connue, la biologie de ce genre est absolument unique chez les puces puisque la femelle s’introduit totalement dans le derme ou l’épiderme de son hôte, y est fécondée, transforme son abdomen en “usine à oeufs”, ou néosome, et passera sa vie à pondre. Il nous semble indiscutable que ces faits surprenants, impliquant chez *Tunga* des adaptations non seulement de la morphologie des mâles comme des femelles, mais aussi de leur éthologie et de leur écologie, méritent de s’y attarder, et ceci nous amènera dans une autre note à discuter de la place des “chiques” dans la systématique des Siphonaptères.

Le spectre parasitaire du genre *Tunga* est également curieux avec une majorité vivant aux dépens des rongeurs (sept espèces), les autres se tournant soit vers les Xénarthres, mammifères primitifs (trois espèces), soit vers l’homme et divers animaux de taille moyenne ou grande, essentiellement des animaux synanthropes (deux espèces). Il faut souligner que, quel que soit l’hôte, grand mammifère ou petit rongeur, la taille de l’espèce-parasite est identique, de l’ordre de 0,9 à 1 mm, quel que soit le sexe, en dehors des femelles enkystées ou néosomiques, bien sûr.

L’exégèse des anciennes publications nous montre toutefois que, en plus de *T. penetrans* sur laquelle existe une littérature pléthorique (voir en particulier Guyon, 1870), quatre autres puces de ce genre avaient été observées au XIXème siècle, mais non décrites : trois au Brésil correspondant très vraisemblablement, d’après les hôtes ou la localisation des néosomes, à *T. caecata*, *T. bossii*, de description récente, et *T. bondari* (*in* Burmeister, 1853 et 1854, et *in* Heusser & Claraz, 1860, auteurs cités par Smit, 1962a) ; la quatrième fut notée par Blandford, (1894) sur des rats d’égouts à Ningpo, en Chine, et correspond, avec une quasi certitude, à *T. caecigena*.

## Répartition, hôtes et descriptions

**Fig. 1.**
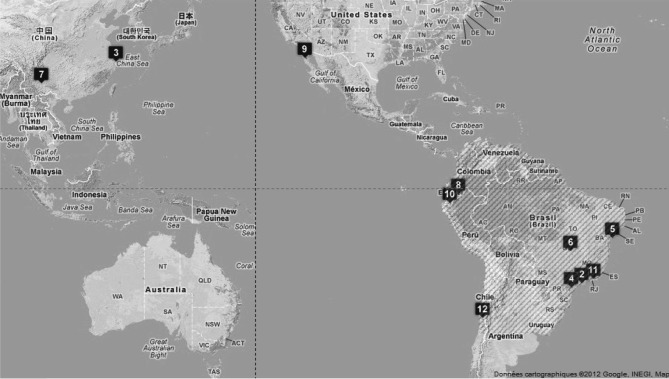
– Carte de la répartition des 12 espèces décrites dans le genre *Tunga* Jarocki, 1838. Chaque chiffre correspond à la localité type d’une espèce. Les espèces sont classées de 2 à 12 en fonction de leur ordre chronologique de description. Le numéro 1, qui revient à *T. penetrans*, ne peut être valablement précisé. La zone en grisé correspond à l’aire que nous supposons primitive, mais le Brésil est le pays désigné par Linné.

Nous décrirons les espèces par ordre chronologique, en précisant la localité type, les autres pays ou régions concernés (figure 1), la synonymie, le groupe biologique, les hôtes et l’état actuel des descriptions (femelle et/ou mâle, larve éventuellement).

Des 12 taxons actuellement décrits, neuf sont, au moins primitivement, néotropicaux, un est néarctique et deux sont paléarctiques. Toutefois, *Tunga penetrans* qui est actuellement aussi abondante en région afrotropicale, zone conquise, qu’en région néotropicale, zone primitive, pose un petit problème. L’hôte ou les hôtes primitifs de cette espèce ne semblent guère discernables.

Par ailleurs, si l’on admet l’invasion du continent américain, à partir de la région paléarctique, par des rongeurs passant par le détroit de Béring (*cf. inter alia* Patterson, 1957), on ne peut que constater qu’en dépit de la prééminence du nombre d’espèces de *Tunga* en région néotropicale, tout se passe comme si les deux taxons connus de Chine représentaient les formes ancestrales du genre. Ceci fut implicitement reconnu par Smit, (1962a) qui divisa ce genre en deux groupes, celui qui renferme les espèces chinoises étant classé comme le plus “primitif”.

### 1 – *Tunga Penetrans* (Linné, 1758)

Localité type : “Brésil” *teste* Linné, 1758.

Autres pays ou régions concernés : toute l’Amérique du Sud, du nord de l’Argentine Macchiavello, (1948) la cite pour le Chili “*à partir de l’île de Chiloë*”), au Mexique où cette espèce, autrefois considérée comme un fléau, semble actuellement ignorée (Staden, 1557; Vizy, 1863 ; Karsten, 1865 ; Bonnet, 1867 ; Blanchard, 1890 ; Macchiavello, *op. cit.* ; Hopkins & Rothschild, 1953 ; Linardi & Guimarães, 2000 ; Acosta et al., 2008...). Les premiers Européens à l’avoir observée semblent être Oviedo en 1526 aux Antilles (Guyon, 1870) et Staden en 1557 au Brésil (Pampiglione & Fioraventi, 2002). Sa présence dans “l’arc antillais” n’est sans doute pas primitive au sens strict, mais elle y est signalée partout dès la colonisation de l’Amérique par les Conquistadores. À l’heure actuelle, elle en a disparu ou est en forte régression un peu partout, cette régression pouvant être cyclique comme à Trinidad, par exemple, où Chadee, (1994) fait état d’un hyperendémisme, alors que Ménier et Mahler (University of West Indies) n’ont pas pu en examiner un seul exemplaire de 2003 à 2005 (*comm. orale*, XII-2011).

*T. penetrans* fut introduite en région afrotropicale, peut-être dès le XVII^ème^ siècle, voire même au XIV^ème^ (Adanson, 1759 ; Hoeppli, 1963). Pour Jeffreys, (1952) “*There is unequivocal evidence for its (Pulex* (sic) *penetrans) presence in Senegambia in 1678 and probable presence at Walata* (actuellement au Mali) *in 1324*”. Certes, on peut admettre que les Phéniciens touchèrent les côtes américaines bien avant l’ère colombienne (Hooton, 1938) et que certains peut-être revinrent vers l’Afrique, mais il nous semble que cette piste demeure fragile. Quoi qu’il en soit, il paraît certain que l’invasion “définitive” du continent eut lieu à la fin du XIXème siècle, en 1872 précisément, à la suite de son introduction en provenance du Brésil, par le sable des ballasts du navire *Thomas Mitchell* touchant terre soit au Gabon, soit en Angola (Blanchard, 1890). Puis, des missions multiples vers l’intérieur du continent à partir de la côte occidentale (explorations, expéditions militaires, constructions de voies ferrées...) “... *amenèrent cette puce vers la côte orientale. En 1891, elle avait traversé le lac Nyassa et pénétré dans l’Ouganda. En 1894, la côte orientale de l’Afrique est atteinte. En 1899, elle est signalée pour la première fois à Madagascar (Blanchard, 1899)”* (Jeanselme & Rist, 1909). Divers auteurs la signalent aux Seychelles, aux Mascareignes, en Inde : “*À la même époque (1899), la chique fut importée dans l’Inde où, le nombre de cas n’a cessé de décroître jusqu’à sa disparition totale... ainsi donc, l’empire indien paraît être à l’abri du fléau*” (Jeanselme & Rist, *op. cit.*). Ces dernières citations correspondent en fait à des implantations temporaires (*cf. inter alia*, Iyengar, 1973). De même, sa collecte en Tunisie, à Tunis précisément (Jordan & Rothschild, 1911), fut le fait d’un apport “touristique” sans lendemain comme il en existe chaque année en tous les points du monde (Hastriter, 1997 ; Degeilh & Beaucournu, 2003). Pour la région afrotropicale, comme pour la région néotropicale, on note localement des pics d’abondance alternant à des périodes de quasi-disparition ; on peut citer, par exemple, le cas de Madagascar (Beaucournu & Fontenille, 1993).

Synonymie :

- *Pulex minimus cutem penetrans* Catesbay, 1743 (dénomination pré-linnéene)
- *Pulex minutissimus nigricans* Barrère, 1743 (idem)
- *Acarus fuscus sub cutem nidulans* Brown, 1756 (idem)
- *Pulex penetrans* Linné, 1758 maregr & piso, bras., Tunga
- *Pulex reptans* Illiger, 1805
- *Rhynchoprion penetrans* (Linné), Oken, 1815
- *Tunga penetrans* (Linné), Jarocki, 1838
- *Dermatophyllus penetrans* (Linné), Lucas, 1839
- *Dermatophylus penetrans* (Linné), Lucas, 1839
- *Sarcophaga penetrans* (Linné), Westwood, 1840
- *Sarcopsylla penetrans* (Linné), Westwood, 1840
- *Sarcopsylla canis* Westwood, 1840 ? *nomen nudum*
- *Dermatophilus penetrans* (Linné), Guérin-Méneville, 1843
- *Psammodes penetrans mihi*, Gistel, 1850
- *Sarcopsyllus penetrans* (Linné), Taschenberg, 1880
- *Dermatophilus penetrans* (Linné), Jordan & Rothschild, 1906
- *Tunga penetrans* Linné (1758), Jordan & Rothschild, 1921
- *Tunga (Tunga) penetrans* (Linné), Lewis, 2009

Groupe : *penetrans* (pour cette classification en “groupes”, *cf*. paragraphe “Phylogénie”).

Hôtes (nous suivrons Wilson & Reeder (1993) pour le nom des hôtes et leur place dans la classification) : *Homo sapiens* (hôte type) ; *Canis lupus* (= *familiaris*) ; *Canis* sp. ; *Felis catus* (Carnivora) ; *Sus scrofa* ; *Bos taurus* ; *Bos* sp. ; *Capra hircus* ; *Ovis aries* ; *Equus caballus* ; *Equus* spp. ; *Tapirus americanus* ; *Pecari tajacu* ; *Lama glama* ; *Vicugna vicugna* (Artiodactyla) ; *Dasypus hybridus* ; *Dasypus* sp. ; *Chaetophractus villosus* ; *Tamandua tridactyla* (Xénarthres) ; *Rattus rattus* ; *R. norvegicus* ; *Mus musculus* (Rodentia, Muridae, Murinae) ; *Cavia* sp. (Rod., Caviidae) (Macchiavello, 1948 ; Hopkins & Rothschild, 1953 ; Linardi & Guimarães, 2000 ; Ezquiaga *et al.*, 2008 ; Beaucournu, non publié). Les références concernant les rongeurs sont pour la plupart extraites de Macchiavello et nous ne pouvons ni les confirmer, ni les infirmer. Macchiavello (*op. cit.*) ajoute le gallinacé *Gallus gallus*, puis Linardi & Guimarães (2000) citent un autre oiseau, le passeriforme *Volatina jacarina* ou Tiziu. Il s’agit d’hôtes manifestement très accidentels, si tant est qu’il n’y ait pas eu erreur d’identification soit de l’hôte, soit du parasite : à plusieurs reprises, *Hectopsylla* (sans doute *psittaci* Frauenfeld, 1860) fut confondue avec *Tunga*.

En région afrotropicale, l’homme est de loin l’hôte le plus souvent cité et, par exemple, Hopkins & Rothschild (1953) ne citent cette puce pour le continent africain et Madagascar que de l’homme ou de ses habitations. Les divers auteurs ayant rédigés une liste faunistique pour cette région ou une partie de celle-ci (De Meillon *et al.*, 1961 ; Lumaret, 1962 ; Haeselbarth, 1966 ; Beaucournu & Fontenille, 1993 ; Segerman, 1994 ; Beaucournu, 2004) se contentent de signaler sa présence sur l’homme et quelques animaux domestiques. Toutefois, Ribeiro (1974) cite en Angola une femelle sur *Potamochoerus porcus* (Suidé) ; de même, nous avons identifié en République Démocratique du Congo une femelle non gorgée sur *Hybomys univittatus* (Muridé) (Beaucournu, non publié).

État actuel des descriptions : mâle, femelles libre et enkystée sont connues : Jordan (1948) ; Traub (1950) ; Hopkins & Rothschild (1953). La larve fut d’abord décrite par Bonnet (1867), puis une étude plus approfondie en fut donnée par Hicks (1930).

### 2 *Tunga Caecata* (Enderlein, 1901)

Localité type : Piracicaba (22° 43’ S – 47° 38’ O), São Paulo, Brésil.

Autres régions concernées : provinces de Minas Gerais (Ouro Preto : 20° 23’ S – 43° 40’ O) et de Paraná (Curitiba : 25° 25’ S – 49° 15’ O), Brésil (Linardi & Guimarães, 2000).

Synonymie :

- *Sarcopsylla caecata* Enderlein, 1901
- *Dermatophilus caecata* (Enderlein), Jordan & Rothschild, 1906
- *Tunga caecata* Enderl. (1901), Jordan & Rothschild, 1921
- non *Tunga caecata* Enderlein, in Banks, 1964, *err. det.*
- *Tunga (Brevidigita) caecata* (Enderlein), Lewis, 2009

Groupe : *caecata*.

Hôtes : *Rattus rattus* (hôte type) ; *R. norvegicus* ; *Mus musculus* (Rod., Muridae, Murinae) ; *Akodon cursor* ; *Rhipidomys mastacalis* ; *Nectomys squamipes* (Rod., Muridae, Sigmodontinae).

État actuel des descriptions : le mâle est toujours inconnu, de même que la femelle libre (Beaucournu *et al.*, 2012). La femelle enkystée a été sommairement décrite par Enderlein (1901), puis *in* Hopkins & Rothschild (1953) et enfin par Linardi & Guimarães (2000). La première observation vraisemblable de ce taxon remonte à Burmeister (1853) qui écrit, à propos de son expédition au Brésil “*... erhielt einmal eine Maus, die an dem einen Ohr 13, am andern 14 grosse Sandflöhe under der Haut beherbergte*” (texte cité par Smit, 1962a).

### 3 *Tunga Caecigena* Jordan & Rothschild, 1921

Localité type : Ning-Po (29° 52’ N – 121° 31’ E), Chekiang, Chine.

Autres régions ou pays concernés : Shangaï (31° 15’ N – 121° 28 E) ; Dong Chia Hong près de Soochow (31° 18’ N – 120° 37’ E) ; Kiangsu (33° 00’ N – 120° 00’ E) ; Dong Chia Hong près de Soochow, Kiangsu ; Wu Tung Chiao (29° 26’ N – 103°51’ E), Szechuan ; Foochow (26° 06’ N – 119° 17’ E), Fukien ; Futsing, Fukien ; toutes ces localités en Chine (Smit, 1962b *in* Jordan, 1962). Introduite au Japon : Osaka (34° 30’ N – 135° 30 E) ; Nishinomiya, près de Kôbe (34° 41’ N – 135° 10’ E) ; Honshu (36° 00’ N - 138° 00’ E) (Smit, 1962b *in* Jordan, 1962 ; Sakaguti & Jameson, 1962).

Synonymie :

- *Dermatophilus lagrangei* Roubaud, 1925
- *Tunga (Brevidigita) caecigena* Jordan & Rothschild, Wang, 1976

Groupe : *caecata*.

Hôtes : *Rattus norvegicus* (hôte type) ; *R. rattus* ; *R. losea* ; *Mus musculus* ; *Mus bactrianus* (Rod., Muridae, Murinae) ; *Suncus murinus* (Insectivora, Crocidurinae).

État actuel des descriptions : la femelle enkystée fut d’abord décrite (Jordan & Rothschild, 1921 ; Roubaud, 1925), puis la femelle libre (Jordan, 1962) ; le mâle a été étudié par Chen & Ku (1958), puis par Sakaguti & Jameson (1962).

### 4 *Tunga Travassosi* Pinto & Dreyfus, 1927

Localité type : Sorocaba (23° 29’ S – 47° 27’ O), São Paulo, Brésil.

Autre région concernée : province de Minas Gerais (localité non précisée), Brésil (Linardi & Guimarães, 2000).

Synonymie :

- *Tunga (Tunga) travassosi* Pinto & Dreyfus, Lewis, 2009

Groupe : *penetrans*.

Hôte : *Dasypus novemcinctus* (Xénarthre, Dasypodidae).

État actuel des descriptions: seule la femelle enkystée est connue (Pinto & Dreyfus, 1927 ; Hopkins & Rothschild, 1953 ; Linardi & Guimarães (2000).

### 5 *Tunga Bondari* WAGNER, 1932

Localité type : Bahia, Salvador (12° 59’ S – 38° 31’ O), Brésil.

Autres régions concernées : provinces de Minas Gerais (Serra da Canastra : coord. ?) et de São Paulo (Franca : 20° 32’ S – 47° 24’ O), Brésil (Hopkins & Rothschild, 1953 ; Linardi & Guimarães, 2000).

Synonymie :

- *Tunga (Tunga) bondari* Wagner, Lewis, 2009

Groupe : *penetrans*.

Hôtes : *Tamandua tetradactyla* (Xénarthre, Myrmecophagidae).

Le Sériema, ou Cariama huppé (*Cariama cristata*, Cariamidae), oiseau cité par Hopkins & Rothschild (1953) et repris sans commentaire par Linardi & Guimarães (2000), nous semble totalement accidentel, vraisemblablement fruit d’une erreur de collecte ou d’étiquetage.

État actuel des descriptions : seule la femelle enkystée est connue (Wagner, 1932 ; Hopkins & Rothschild, 1953 ; Linardi & Guimarães, 2000).

### 6 *Tunga Terasma* Jordan, 1937

Localité type : Anápolis (16° 20’ S – 48° 58’ O), Goyaz, Brésil.

Autres régions concernées : provinces de Minas Gerais (Serra da Canastra : coord.? ; Unai : 16° 23’ S – 46° 53’ O) et de São Paulo (localité non précisée), Brésil (Linardi & Guimarães, 2000).

Synonymie :

- *Tunga travassosi* Pinto & Dreyfus, *teste* Fonseca, 1936, *err. det.*
- *Tunga (Tunga) terasma* Jordan, Lewis, 2009

Groupe : *penetrans*.

Hôtes : *Cabassous unicinctus* (hôte type) ; *Euphractus sexcinctus* ; *Priodontes maximus* (Xénarthres, Dasypodidae).

État actuel des descriptions : le mâle et la femelle enkystée sont connus (Hopkins & Rothschild, 1953 ; Linardi & Guimarães, 2000).

### 7 *Tunga Callida* Li & Chin, 1957

Localité type : province du Yunnan, Chine.

Autres régions concernées : “South west mountain subregion” et “West plateau montain subregion” (Liu Zhiying *et al.*, 1986). Cette dernière région est commune avec *T. caecigena*.

Synonymie :

- *Tunga (Brevidigita) callida* Li & Chin, 1957 ; Wang, 1976

Groupe :*caecata*.

Hôtes : *Rattus* sp. (hôte primaire) ; *Rattus rattus* ; *R. norvegicus* ; *Apodemus chevrieri* ; *Mus bactrianus* (Rod., Muridae, Murinae) ; *Eothenomys custos* (Rod., Arvicolidae).

État actuel des descriptions : la femelle enkystée (Li & Chin, 1957), puis le mâle (Wang, 1976) sont connus.

### 8 *Tunga Libis* Smit, 1962

Localité type : Riobamba (1° 40’ S – 78° 38’ O), Chimborazo, Équateur.

Autres régions concernées : Whiting *et al.* (2008) citent *Tunga libis* sur *Phyllotis andium*. Il s’agit de deux femelles récoltées au Pérou, région d’Ancash (9° 30’ S – 77° 45’ O), mais dont l’identification, bien que très vraisemblable, n’est plus certaine à la lumière de la description de *T. bonneti* (Hastriter, *in litt.* 18.01.12).

Synonymie :

- non *T. libis* Smit, femelles signalées du Chili, *in* Smit, 1969, *err. det.* pour *T. bonneti*
- *Tunga libis*, *in* Whiting *et al.*, 2008 ?
- *Tunga (Tunga) libis* Smit, Lewis, 2009

Groupe : *caecata*.

Hôtes : *Akodon mollis* (hôte type) ; *Phyllotis andium* ? (Rod., Muridae, Sigmodontinae).

État actuel des descriptions : seul le mâle holotype est connu avec certitude (Smit, 1962a *cf.* Beaucournu *et al.*, 2012). Une ou deux femelles sont signalées du Pérou (Whiting *et al.*, 2008), mais elles ne furent pas décrites et, selon Hastriter (*in litt.*, II. 2012), cette détermination est à vérifier depuis la découverte de *bonneti*, qui est proche de *libis*. Le néosome est situé dans le pavillon de l’oreille comme chez *caecata* et *monositus*, pour ne citer que des taxons “américains” ; la spermathèque, sphérique, peut évoquer celle de *bossii*.

### 9 *Tunga Monositus* Barnes & Radovsky, 1969

Localité type : San Quintin Bay (30° 27’ N – 116° 12’ O), Baja California, Mexique.

Autres régions ou pays concernés : San Martin Island, Baja California, Mexique ; Zion National Park (37° 10 N – 113° 0’ O), Washington County, Utah, USA (Hastriter, 1997).

Synonymie :

- non *Tunga caecata* Enderlein, *in* Banks, 1964, *err. det.*, *cf.* Barnes & Radovsky, 1969
- *Tunga (Tunga) monositus* B. & R., Lewis, 2009

Groupe : *caecata*.

Hôtes : *Peromyscus maniculatus* (hôte type) ; *P. eremicus* ; *P. crinitis* ; *Neotoma lepida* ; *Neotoma* spp. (Rod., Muridae, Sigmodontinae).

État actuel des descriptions : mâle, femelles libre et enkystée sont connus, de même que la larve qui présente la particularité par rapport à *penetrans* (seule autre *Tunga* chez qui la larve a été décrite) d’avoir des pièces buccales atrophiées (Barnes & Radovsky, 1969).

### 10 *Tunga Trimamillata* Pampiglione, Trentini, Fioraventi, Onore & Rivasi, 2002

Localité type : Santa Isabel (3° 21 S’ – 79° 19’ O), Azuai, Équateur.

Autres régions ou pays concernés : Équateur : province de Loja, (4° 0’ S – 79° 13’ O), (Pampiglione *et al.*, 2004), Machala (3° 16’ S – 79° 58’ O) (Pampiglione/ Fioraventi, non publié) ; Pérou : Piura et Suyo, province de Ayabaca (4° 39’ S – 79° 40’ O) (Fioraventi *et al.*, 2006) ; Brésil (*sine loco*) (Eiseli *et al.*, 2003 ; Linardi, *in litt.*).

Synonymie :

- *Tunga (Tunga) trimamillata* Pampiglione *et al.*, Lewis, 2009

Groupe : *penetrans*.

Hôtes : *Capra hircus* (hôte type) ; *Bos taurus* ; *Sus scrofa* ; *Ovis aries* (Artiodactyles) ; *Homo sapiens*.

L’hôte type n’est pas indiqué par les auteurs qui écrivent “*This makes T. trimamillata more similar to T. penetrans which is the only one among the nine species known of genus Tunga not limited to a single or to a few closely related host species*” (Pampiglione *et al.*, 2002). Toutefois, Linné a bien spécifié que l’homme était l’hôte de *Pulex penetrans*. Étant donné que Pampiglione *et al.* précisent dans leurs deux premières publications sur cette espèce (2002, 2003) que leur nouvelle *Tunga* parasitait “*goats, swine and cattle*” ou “*goats, pigs and cattle*”, nous pensons pouvoir désigner comme hôte type la chèvre, *Capra hircus*. Actuellement, à notre connaissance, aucun mammifère indigène n’est signalé.

État actuel des descriptions : mâle, femelles libre et enkystée sont décrits (Pampiglione *et al.*, 2003 ; Pampiglione *et al.*, 2004).

### 11 *Tunga Bossii* Avelar De, LinharèS & Linardi, 2012

Localité type : Serra de Itatiaia, Itatiaia National Park (22° 26’ S – 44° 51’ O), Rio de Janeiro, Brésil.

Synonymie : néant.

Groupe : *caecata*.

Hôte : *Delomys dorsalis* (Rod., Muridae, Sigmodontinae). État actuel des descriptions : seule la femelle holotype enkystée est connue (Avelar *et al.*, 2012). Cette espèce avait été vue, mais non décrite, dans la province de Minas Gerais, par Reinhardt en 1853 (*teste* Burmeister, 1854, cité et commenté par Smit, 1962 qui écrit : “*Mesomys spinosus* (ce taxon ne nous est pas connu) *suffers from sand-fleas which preferably burrow near the anus and genitalia, at the base of the tail. This is very reminiscent of the habit of the recently described chinese Tunga callida and I wonder whether indeed a similar species does occur in Brazil*”). Dans le même paragraphe, Burmeister nous informe que le Dr Reinhardt “*Zeigte mir in Lagoa Santa (Minas Gerais) eine Hausmaus (= Mus musculus), die 13 solcher Flöhe an dem einen und 12 am andern Ohr hatte*”. Cette fois, il ne peut s’agir que de *T. caecata*. Burmeister puis Reinhardt auraient donc, apparemment, vu les deux *Tunga* inféodées aux rongeurs dans ce pays, *caecata* et *bossii* !

### 12 *Tunga Bonneti* Beaucournu & Gonzálezacuña, 2012

Localité type :Quebrada Higuera (31° 30’ S – 71° 06’ O), Atacama, Chili (holotype).

Autres régions concernées du Chili :provinces de Santiago (33° 29’ S – 71° 37’ O) ; Linari (30° 38’ S – 71° 31’ O) ; Huasco (28° 02’ S – 71° 06’ O) ; Chañaral (26° 09’ S – 70° 40’ O) ; Antofagasta (25° 24’ S – 70° 30’ O) ; El Loa (21° 10’ S – 68° 19’ O) (allotype et paratypes).

Synonymie :

- *Tunga libis* Smit, 1962a, *in* Smit, 1969, femelles du Chili, *err. det.*

Groupe : *caecata*.

Hôtes : *Phyllotis darwini* (hôte type) ; *Ph. xanthopygus* (Rod., Muridae, Sigmodontinae).

État actuel des descriptions : le mâle et les femelles libre et enkystée sont connus (Beaucournu *et al.*, 2012).

### 13 *Tunga* Sp. Nova Avelar *et al.* (Sous Presse)

Aucun document n’est encore disponible, sauf l’origine : Brésil (Linardi, *in litt.*, 2012).

## Phylogénie

La proposition de Smit (1962a), basée tant sur l’anatomie que sur la spécificité, de diviser *Tunga* en groupes est excellente car elle permet de mieux appréhender ce genre. En revanche, et nous sommes en plein accord avec Linardi & Guimarães (2000), la scission taxonomique de Wang (1976) qui créait le sous-genre *Brevidigita* pour les deux taxons paléarctiques, *caecigena* et *callida*, nous semble sans valeur, en dépit de son acceptation par Lewis (2009) qui y ajoutait *caecata*.

Smit (*op. cit.*) scinde le genre *Tunga* en deux groupes, et ceci sur des critères parasitaires et morphologiques. Nous le citons :

- “Penetrans*-group – Parasites of South American Edentates...* T. penetrans*, however is promiscuous in the choice of host... This is the advanced, or degenerate, group. The pronotum is dorsally completely fused with the mesonotum and the chaetotaxy of the fifth tarsal segment is strongly reduced, there being only two pairs of hair-like lateral plantar setae present and no patch of minute plantar setae; in the female the spiracular fossae of terga II-IV have vanished in* T. penetrans *(this character could not be checked in the other species of this group):* penetrans*,* bondari*,* travassosi*, and* terasma”. (*trimamillata* ne fut isolée qu’en 2002).
- “Caecata*-group – Parasites of murid rodents (*Rattus*,* Mus*,* Akodon*). This is the primitive group. The pronotum is dorsally not, or not wholly, fused with the mesonotum and the chaetotaxy of the fifth tarsal segments is not much reduced, there being three or four pairs of stiff subspiniform lateral plantar setae as well as a patch of minute plantar setae; in the female the spiracular fossae of terga II-IV are small in* T. caecigena *(the available material of the other species in this group does not show terga II-IV or not clearly):* caecata*,* libis*,* caecigena*, and* callida”. (*monositus* ne fut décrite qu’en 1969 ; s’y sont ajoutées depuis *bossii* et *bonneti*, ces deux *Tunga* étant décrites en 2012).

À l’opposé de ce que montrent *T. penetrans* et *T. trimamillata*, parasites peu spécifiques s’attaquant essentiellement à des moyens ou grands mammifères, particulièrement l’homme pour *penetrans*, les espèces de *Tunga* liées aux Xénarthres (*T. travassosi*, *T. bondari*, *T. terasma*) sont certes peu connues, mais la liste donnée ci-dessus nous a montré un tropisme très net : Dasypodidae pour *travassosi* et *terasma*, Myrmecophagidae pour *bondari*. Le fait que les Megalonychidae et les Bradypodidae ne soient, apparemment, pas parasités vient vraisemblablement de leurs moeurs arboricoles.

Ce sont les rongeurs Muridae qui représentent la famille hôte primitive des *Tunga*, hébergeant sept espèces sur les 12 connues. Les Muridae-Sigmodontinae (*ex* Cricetidae *pro parte*) ont explosé en Amérique, c’est-à-dire dans les régions néarctique et néotropicale : les Sigmodontinae dérivent d’un stock commun avec les Murinae et leur répartition y couvre les zones équatoriales et tempérées chaudes correspondant à la dispersion des *Tunga* du groupe *caecata*. Dans cette optique, on doit admettre que les deux espèces paléarctiques connues de Chine, *T. caecigena* et *T. callida* (introduite au Japon pour *T. caecigena*) sont réellement, et primitivement, parasites de Murinae, *Rattus* spp., essentiellement.

Les espèces néotropicales, ou du sud de la région néarctique pour *T. monositus*, sont toutes liées aux Sigmodontinae ; d’ailleurs, les Murinae ont tous été introduits dans cette région biogéographique. Linardi & Guimarães (2000) considéraient encore que *T. caecata* était inféodée aux Murinae (*Rattus* spp. et *Mus*). Il faut attendre un article récent de Avelar & Linardi (2010) pour voir que cette puce est seulement un peu moins spécifique que les autres *Tunga* néotropicales, ou néarctiques, de ce groupe : les rats sont encore cités, mais divers Sigmodontinae sont notés comme hôtes.

## Chorologie, phénologie, écoologie, sexe-ratio

Nous pensons avoir montré (Beaucournu *et al.*, 2012) que les sols pulvérulents, donnés comme favorables pour *T. penetrans* par la majorité des auteurs, à l’exception de Bonnet (*op. cit.*) qui ne mentionne pas de milieux favorisants, l’étaient également pour les autres espèces congénériques. De plus, les biotopes “ouverts” semblent être préférés, voire privilégiés par *Tunga*. À notre avis, la latitude mise à part, car son incidence par le biais de la température est certaine, la plupart des autres paramètres sont négligeables. L’altitude, par exemple, ne semble curieusement jouer aucun rôle en dépit de son implication dans la température ambiante. *T. penetrans* fut collectée en abondance à 2 661 m d’altitude à Santa Fé de Bogota (Hoeppli, 1963) et jusqu’à 3 100 m dans un autre point, non précisé, de Colombie (Guyon, 1870). L’holotype de *T. libis*, en Équateur, a été récolté à 2 200 m ; les types de *T. monositus* furent collectés au niveau de la mer. Enfin, si l’allotype de *T. bonneti* fut également noté à ce niveau, l’un des paratypes vient de 3 794 m !

Roubaud (1925) est le premier à avoir abordé la phénologie des *Tunga* à propos de *Dermatophilus lagrangei* (= *Tunga caecigena*) : “*C’est pendant la saison froide, de janvier à mars, que les rats porteurs de tumeurs à chiques ont été rencontrés... Tous les parasites sont sensiblement au même stade de développement, ce qui semble indiquer que l’évolution parasitaire de* Dermatophilus lagrangei *est très saisonnière*”. Pour cette même espèce, (Smit, 1962b *in* Jordan, 1962) écrit “*The fleas are probably univoltine and are usually found in the cold season... The closely related* Tunga callida *Li and Chin, 1957, also, has been collected only during the period November to March*”. Les données publiées par Barnes & Radovsky (1969), puis par Hastriter (1989) à propos de *T. monositus*, et enfin celles de Beaucournu *et al.* (2012) basées sur un nombre très important d’observations de néosomes de *T. bonneti*, vont dans le même sens. Notons toutefois que *T. bossii*, du Brésil, fut collectée en décembre.

Chez les Siphonaptères, la vie larvaire la plus classique nous montre des larves évoluant dans la litière de l’hôte, en même temps le plus souvent que les mammifères ou oiseaux juvéniles. Les larves se nourrissent de débris protéiniques et impérativement de sang, celui-ci provenant des *excreta* des puces adultes hématophages. Divers genres ou espèces de puces, *Xenopsylla* (Pulicidae) par exemple, dont les hôtes vivent en terrier, vont se rencontrer dans le sol à très fine granulométrie. C’est cette option que les *Tunga* ont adoptée, mais chez *T. monositus*, au moins, la larve dépourvue de pièces buccales ne se nourrit pas.

L’écologie des *Tunga* adultes est bien sûr originale. Les deux sexes se nourrissent, mais le mâle à la surface du corps de l’hôte (c’est un ectoparasite), et la femelle à l’intérieur de celui-ci (c’est un mésoparasite). Mâles et femelles, bien que montrant une troisième paire de pattes plus longue que les deux premières, donc théoriquement “adaptée au saut”, sont connus, au moins chez *penetrans* et *trimamillata*, pour être de mauvais sauteurs. Ceci n’est d’ailleurs pas exceptionnel chez les Siphonaptères et se rencontre dans des genres ou familles très divers : chez *Glaciopsyllus* (Ceratophyllidae) et *Parapsyllus* (Rhopalopsyllidae), ces deux genres parasitant des oiseaux ; chez les Ischnopsyllidae qui sont inféodés aux chauves-souris, et bien d’autres... Fait unique chez les Siphonaptères, et observé dès 1865 par Karsten, le mâle dans le genre *Tunga* est placé pendant la copulation au-dessus de la femelle (ce qui est classique chez les insectes) et non en dessous comme chez toutes les autres puces : cette caractéristique fut notée par divers entomologistes “anciens” (Roesel von Rosenhof, 1749 chez *Ctenocephalides* ; Karsten, *op. cit.* chez *Pulex irritans*). Notons enfin que l’oeuf n’est pas simplement pondu, mais expulsé à quelques millimètres du corps de l’hôte.

En corollaire à la position du mâle pendant la copulation, le phallosome est unique dans sa morphologie. Chez toutes les autres puces, le “corps” de cet organe, ou phallosome, est “monolithique” comme d’ailleurs, et c’est un fait curieux, chez les insectes à accouplement dorso-ventral. Chez *Tunga*, il est articulé, pouvant se plier à environ 45° : cette adaptation permet l’intromission de l’organe dans les voies génitales de la femelle, voies qui par l’enkystement total de celle-ci sont orientées à 90° de l’axe du corps du mâle posé, lui, sur la peau de l’hôte.

Bonnet (*op. cit.*) et Guyon (*op. cit.*), en redécrivant *Tunga*, notent que la *coxa* III présente un fort crochet à sa partie antéro-ventrale, crochet qui faciliterait la pénétration de la femelle dans le corps de l’hôte. Cette hypothèse, à première vue séduisante et qui semble confortée par *Neotunga*, est réfutée par le fait que la même conformation se rencontre non seulement chez les mâles de *Tunga*, mâles qui ne sont pas, eux, “mésoparasites”, mais également chez *Echidnophaga* et *Phacopsylla*, toujours “ectoparasites”. L’aptitude au saut chez *Tunga* est faible et est même niée par certains, ce qui est exagéré : son amplitude va de quelques millimètres à quelques centimètres, selon les observations, mais elle existe. Dès que la femelle s’enkyste, l’autotomie de ses pattes débute, commençant par l’apex de la troisième paire. À la phase terminale de l’enkystement, toutes les pattes sont atrophiées, préservant toutefois, au moins, les *coxae*.

Chez les puces sessiles, c’est-à-dire qui sont fixées à vie (*Echidnophaga* ou *Hectopsylla*, par exemple) ou temporairement sur leur hôte par leurs pièces buccales (*Spilopsyllus s.l.* par exemple, c’est-à-dire en incluant les puces décrites dans le genre *Cediopsylla*), celles-ci sont toujours longues et abondamment pourvues de denticulations, comme chez *Tunga*. L’abdomen des femelles, et d’elles seules, montre une augmentation de taille, augmentation mise en évidence par l’aspect blanchâtre des membranes inter-segmentaires distendues et la disposition des tergites et sternites isolés les uns des autres. Ceux-ci ne sont en aucun cas dégénératifs, ni eux, ni les spiracles qu’ils portent.

Parler de sexe-ratio dans le genre *Tunga* où plusieurs taxons se caractérisent par l’extrême rareté des mâles, voire leur absence dans les récoltes (*caecata*, *travassosi*, *bondari*, *bossii*), peut paraître provocateur, mais les raisons de ce déséquilibre doivent exister : chez les Siphonaptères, le sexe-ratio lors des collectes de terrain est, normalement, en faveur des femelles, de l’ordre de 0,6 à 0,9, mais ces chiffres varient selon que l’on étudie les puces prélevées sur l’hôte ou dans son nid, ou dans sa litière, ou enfin après un élevage. Dans certains genres (*Doratopsylla* par exemple), les mâles collectés sur l’hôte sont plus abondants que les femelles, ceci étant lié à une éthologie différente d’un sexe à l’autre.

Chez les puces “classiques”, la présence ou l’absence de l’oeil correspond souvent au mode de vie de l’hôte spécifique, ou de son ectoparasite. La recherche active de l’hôte pousse au bon “développement” de cet organe (ou vice-versa, il faut le reconnaître) : on peut citer *Pulex irritans* (Pulicidae), puce dite “de l’homme”, mais primitivement liée à *Vulpes*, mais aussi *Dorcadia* sp. (Vermipsyllidae), parasite de moyens ou grands artiodactyles (moutons, yacks, chameaux, chevaux...) qui semble pratiquer une “chasse” à l’affut, ou encore *Dasypsyllus* spp. (Ceratophyllidae), puces d’oiseaux de presque toutes les régions du monde, sauf la région afrotropicale, mais dont le nombre de captures sur des petits mammifères, hôtes aberrants, montre que leur quête est sans doute essentiellement visuelle et non “olfactive”. La vie souterraine, ou l’écologie des puces de chiroptères (hôtes et parasites occupant des gîtes obscurs), par l’inutilité de la vue dans ces deux cas de figure, entraîne une atrophie, voire une disparition de l’oeil : citons les Ischnopsyllidae, famille dont tous les genres sont parasites stricts des chauves-souris, ou les *Palaeopsylla* du groupe *minor* (Ctenophthalmidae) inféodées au genre *Talpa*, insectivores strictement souterrains. On peut, au passage, noter que *Peusianapsylla*, sous-genre fossile de *Palaeopsylla*, nous montre des yeux apparemment fonctionnels, ce qui équivaut à dire que les micromammifères hôtes n’avaient pas la biologie des *Talpa* !

Que peut-on tirer de ces observations dans l’étude du genre *Tunga* ? Notons d’emblée que chez les deux espèces du groupe *penetrans* inféodées aux artiodactyles et à l’homme, *T. penetrans* et *T. trimamillata*, les mâles, sans être abondants dans les collectes, ne sont pas rares. Bonnet (*op. cit.*) estime que, *in natura*, le rapport ♀/♂ est de 8/1 chez *penetrans*, ce qui est très faible pour un siphonaptère “normal”, mais semble pléthorique pour une chique ! Bonnet écrit de plus que ce ratio est biaisé par la vie “endoparasitaire” de la femelle. Or, ces deux *Tunga* nous montrent un oeil bien développé. Dans le “sous-groupe” réunissant les trois espèces liées aux xénarthres, seule *T. terasma* possède un oeil pigmenté et grand : c’est la seule chez qui le mâle soit connu. Le groupe *caecata* comporte sept espèces. Le mâle est connu chez *caecigena*, *callida*, *libis*, *monositus* et *bonneti* : l’organe de la vision est pigmenté et développé chez *libis* et *bonneti* ; il est de petite taille chez *monositus* ; il est absent chez *caecigena* et *callida* (comme d’ailleurs chez *caecata* et *bossii*). Il nous semble que nous pouvons suggérer ici un début d’explication : l’oeil, lorsqu’il est présent, va permettre une recherche active de l’hôte, nous l’avons montré ci-dessus. Cette quête se fera à l’air libre pour *penetrans*, *trimamillata* et, sans doute, pour *terasma*, parasite de fourmiliers. Dans le cas des *Tunga* inféodées aux rongeurs, on peut penser que, selon l’acuité visuelle de la puce, la recherche de l’hôte aura lieu soit essentiellement à la surface du sol pour les espèces “bien voyantes” (les deux femelles classées à tort par Smit (1969) comme “*libis*” furent trouvées dans des pièges à formol placés à la surface du sol), soit dans les terriers pour les autres : dans ce cas, le rongeurcible sera détecté par d’autres sens que la vue et le biotope où vit le mâle, essentiellement souterrain, rendra sa capture plus délicate.

## *Dermecos* et rôle pathogène

Rappelons tout d’abord que par “*dermecos*”, terme créé par Smit (1972), il faut entendre “*the microhabitat created by the host-skin and its outgrowths*”. Ici, le *dermecos* est la localisation élective de la femelle enkystée de *Tunga*, c’est-à-dire l’emplacement du néosome.

Les deux espèces de *Tunga* liées aux Artiodactyles (et accessoirement à l’homme pour *T. trimamillata*) semblent se singulariser. *T. penetrans*, mieux étudiée chez l’homme que chez les artiodactyles, est tout particulièrement observée aux niveaux des pieds (sillon périunguéal du gros orteil en particulier) ou des pattes, où le néosome, la classique “chique” ou “boule de gui”, est observable, mais presque toutes les localisations sont possibles. Généralement, le parasitisme est faible (de un à trois par porteur, surtout s’il s’agit d’un touriste), mais chez des autochtones présentant un état clinique invalidant (par exemple, en Afrique intertropicale, une personne en phase terminale de Trypanosomose africaine ou “maladie du sommeil”), le nombre de chiques peut être très élevé, dépassant la centaine. Au XIXème siècle, *T. penetrans* fut une cause grave et fréquente de consultations et/ou d’invalidations temporaires chez les soldats de l’Expédition française au Mexique (Vizy, 1863). Récemment, Chadee (1994), Heukelbach *et al.* (2001 & 2004) ont tracé des listes inquiétantes de maladies pouvant résulter de ce parasitisme dans des communautés défavorisées de Trinidad ou du Brésil. De même, Heymer (1984) insiste sur la lourde incidence de cette puce chez des Pygmées Bayaka sédentarisés au contact des Bantous en forêt tropicale africaine. On ne peut passer sous silence le risque de surinfections par germes telluriques, et en particulier celui du tétanos (*Clostridium tetani*), ceci étant favorisé par les plaies créées au niveau des pieds, soit directement par l’insecte, soit par l’extraction de celuici. Dans un registre moins dramatique, Pampiglione *et al.* (1998) donnent une bonne représentation de la pathologie liée à *T. penetrans* chez le porc à São Tomé ; personnellement, nous avons observé des infestations très élevées au Togo, également chez des porcs, avec plus de 30 chiques par pied !

*T. trimamillata* semble avoir dans les Andes le même *dermecos* que *T. penetrans* (Fioraventi *et al.*, 2006), mais les dimensions des lésions seraient plus grandes et amèneraient davantage de complications, en particulier chez les moutons et les chèvres ; le porc serait moins souvent parasité et, dans ce cas, il y aurait souvent une association parasitaire avec *T. penetrans*, Fioraventi *et al.* (*op. cit.*) notant que les habitants savent différencier les “*niguas de cerro*” (chiques du porc), correspondant à *T. penetrans*, des “*niguas de vaca*” (chiques de vache) qui sont des *T. trimamillata*!

La situation est différente pour les *Tunga* parasites de Xénarthres. Les néosomes sont situés électivement au niveau de la paroi ventrale de l’hôte chez *travassosi* et *terasma* (Hopkins & Rothschild, *op. cit.* ; Linardi & Guimarães, *op. cit.*). Il en est sans doute de même chez *bondari* ? Leur impact sanitaire nous est inconnu.

La localisation des néosomes chez les *Tunga* liées aux rongeurs est variable d’une espèce à l’autre, avec généralement un seul *dermecos* par espèce. Pour *caecata*, *caecigena* et *monositus*, les néosomes se focalisent aux pavillons de l’oreille. On peut penser que Jordan & Rothschild, décrivant *caecigena*, se sont divertis en songeant que si *caecata* était une puce aveugle, *caecigena* rendait son hôte... mal voyant ! Il n’en est rien semble-t-il, même si le lobe de l’oreille retombe et masque l’oeil. Chez *callida*, le *dermecos* est, comme chez *travassosi* et *terasma*, la peau du ventre, mais ici les organes génitaux sont également concernés ; *bossi* semble voisin de *callida* par ces localisations. Pour *bonneti*, enfin, la base de la queue est le site électif, mais c’est sur la face dorsale et non ventrale que l’on peut voir les néosomes et le segment anal de la puce y est bien visible. Chez cette espèce, quelques localisations furent également notées au niveau des oreilles (Beaucournu *et al.*, 2012). Pour *bonneti*, à l’exception d’un hôte qui montrait en aval du néosome une striction de la queue par défaut d’irrigation sur un ou deux centimètres, aucun rongeur parasité ne semblait en souffrir, mais pour cette puce, le nombre de parasites par hôte est faible, un ou deux généralement. Chez *caecata*, *caecigena* et peut-être *callida* et *monositus*, le nombre de néosomes peut dépasser la vingtaine par hôte (*cf. supra*) : leur impact est cependant incertain.

## References

[R1] Acosta R., Fernández J.A., Bousquets J.L. & Jiménez M.C. Catálogo de pulgas (Insecta: Siphonaptera). Catálogo N° 1, Volumen 2. Serie Catálogos del Museo de Zoología “Alfonso L. Herrera”, Universidad Nacional Autónoma de Mexico, Facultad de Ciencias, Mexico D.F, 2008.

[R2] Adanson M. A voyage to Senegal, the Isle of Goree and the river Gambia, 1759 (texte primitivement français, traduit en anglais, non lu), cité par Hoeppli (1963)

[R3] Avelar D.M. & Linardi P.M. Use of multiple displacement amplification as prepolymerase chain reaction (Pre-PCR) to amplify genomic DNA of siphonapterids preserved for long periods in scientific collections. Parasites & Vectors, 2010, 86, 1–6. 10.1186/1756-3305-3-86 PMC2945329 20840790

[R4] Avelar D.M. De, Linharès A.X. & Linardi P.M. A new species of *Tunga* (Siphonaptera: Tungidae) from Brazil with a key to the adult species and neosomes. Journal of Medical Entomology, 2012, 49, 23–28. 22308767 10.1603/me11111

[R5] Banks R.C. Birds and mammals of the voyage of the “Gringa”. Transactions of the San Diego Society of Natural History, 13, 1964, 177–184.

[R6] Barnes A.M. & Radovsky F.J. A new *Tunga* (Siphonaptera) from the nearctic region with description of all stages. Journal of Medical Entomology, 1969, 6, 19–36. 5775471 10.1093/jmedent/6.1.19

[R7] Beaucournu J.C. Catalogue des Puces de la région Afrotropicale (Insecta-Siphonaptera) (sous-région malgache exclue). Beiträge fur Entomologie, 2004, 54, 185–239.

[R8] Beaucournu J.C. & Fontenille D. Contribution à un catalogue des Puces de Madagascar (Insecta, Siphonaptera). Archives de l’Institut Pasteur de Madagascar, 1993, Édition spéciale. 1669363

[R9] Beaucournu J.C. & Horak I.G. *Phacopsylla inexpectata* gen. nov. for *Echidnophaga inexpectata* Smit, 1950 (Siphonaptera, Pulicidae). Journal of African Zoology, 1994, 108, 133–141.

[R10] Beaucournu J.C., Mergey T., Muñoz-Leal S. & Gonzálezacuña D. Description de *Tunga bonneti* n. sp. du Chili (Siphonaptera : Tungidae) et notes sur sa spécificité, sa chorologie, son *dermecos* et sa phénologie. Parasite, 2012, 3, 207–216. 22910663 10.1051/parasite/2012193207 PMC5394828

[R11] Blanchard R. Traité de zoologie médicale. J.B. Baillière & Fils, Paris, 1890, tome 2, 484–493.

[R12] Blanchard R. Présence de la chique (*Sarcopsylla penetrans*) à Madagascar. Archives de Parasitologie, 1899, 2, 627–630.

[R13] Blandford W.F.H. The chigoe in Asia. Entomologist’s Monthly Magazine, 1894, 5, 228–230.

[R14] Bonnet G. Mémoire sur la puce pénétrante ou chique (*Pulex penetrans* L.). J.B. Baillière & Fils, Paris, 1867.

[R15] Burmeister H.C.C. Reise nach Brasilien, durch die Provinzen von Rio de Janeiro und Minas Geraës. Berlin, 1853.

[R16] Burmeister H.C.C. Systematische Uebersicht der Thiere Brasiliens, welche während einer Reise duch die Provinzen von Rio de Janeiro und Minas Geraës gesammelt oder beobachtet wurden. Berlin, 1854.

[R17] Chadee D.D. Distribution patterns of *Tunga penetrans* within a community in Trnidad, West Indies. Journal of Tropical Medicine and Hygiene, 1994, 97, 167–170. 8007057

[R18] Chen C.S. & Ku H.D. The discovery of a male specimen of the sandflea, *Tunga caecigena* Jordan & Rothschild, 1921 with a morphological description (en chinois). Acta Entomologica Sinica, 1958, 8, 179–184.

[R19] Degeilh B. & Beaucournu J.C. Tungose, *in*: Parasitoses et mycoses courantes de la peau et des phanères. Elsevier SAS, 2003, 55–63.

[R20] De Meillon B., Davis D.H.S. & Hardy F. Plague in Southern Africa. Volume I: The Siphonaptera. Government Printer, Pretoria, 1961.

[R21] Doby J.M. Des compagnons de toujours. I - La Puce. Chez l’auteur, L’Hermitage (Ille-et-Vilaine), 1996.

[R22] Doucet J. Tungose, *in*: Encyclopédie Médico-Chirurgicale, Maladies infectieuses, Paris, 1969, 8120 D10.

[R23] Eiseli M., Heukelbach J., Matck Van E., Mehlhorn H., Meckes O., Franck S. & Feldmeier H. Investigations on the biology, epidemiology, pathology and control of *Tunga penetrans* in Brazil: I. Natural history of tungiasis in man. Parasitological Research, 2003, 90, 87–99. 10.1007/s00436-002-0817-y 12756541

[R24] Enderlein G. Zur Kenntnis der Flöhe und Sandflöhe. Neue und wenig bekannte Pulliciden und Sarcopsyllidae. Zoologische Jahrbücher, 1901, 14, 549–557.

[R25] Ezquiaga M.C., Lareschi M., Abba A.M. & Navone G.T. Nuevos registros de Pulgas (Siphonaptera) parásitas de Dasipódidos (Mammalia: Xenarthra) en el noreste de la provincia de Buenos Aires, Argentina. Mastozoología Neotropical, 2008, 15, 193–196.

[R26] Fioraventi M.L., Pampiglione S. & Trentini M. A second species of *Tunga* (Insecta: Siphonaptera) infecting man: *Tunga trimamillata*. Parasite, 2003, 10, 282–284. 14535170

[R27] Guyon M.J.L.G. Histoire naturelle et médicale de la chique, *Rhynchoprion penetrans* (Oken). Chez l’auteur, Paris, 1870.

[R28] Haeselbarth E. Siphonaptera, *in*: The Arthropod parasites of Vertebrates in Africa south of the Sahara. The South African Institute for Medical Research, Johannesburg, 1966, vol. 13 n° 52, 117–212.

[R29] Hastriter M.W. Establishment of the Tungid Flea, *Tunga monositus* (Siphonaptera: Pulicidae), in the United States. Great Basin Naturalist, 1997, 57, 281–282.

[R30] Heukelbach J., Franck S. & Feldmeier H. High attack rate of *Tunga penetrans* (Linnaeus 1758) infestation in an impoverished Brazilian community. Transactions of the Royal Society of Tropical medicine and Hygiene, 2004, 98, 431–434. 15138080 10.1016/j.trstmh.2003.12.004

[R31] Heukelbach J., Sales De Oliveira F., Hesse G. & Feldmeier H. Tungiasis: a neglected health problem of poor communities. Tropical Medicine and International Health, 2001, 6, 267–272. 11348517 10.1046/j.1365-3156.2001.00716.x

[R32] Heusser J.C. & Claraz G. Thierleben in der Brasilianischen Provinz Rio de Janeiro. Pettermann’s Mitteilungen, 1860, 7, 247–257.

[R33] Heymer A. Sédentarisation, acculturation et maladies infectieuses, un problème socio-écologique des Pygmées Bayaka. Bulletin de la Société de Pathologie exotique, 1985, 78, 226–238. 4028313

[R34] Hicks E.P. The early stages of the Jigger, *Tunga penetrans*. Annals of Tropical Medicine and Parasitology, 1930, 24, 575–586.

[R35] Hoeppli R. Early References to the Occurrence of *Tunga penetrans* in Tropical Africa. Acta Tropica, 1963, 20, 143–153. 13963854

[R36] Hooton E.A. Apes, men and morons. New York, 1938 (cité par Jeffreys, 1952).

[R37] Hopkins G.H.E. & Rothschild M. An illustrated catalogue of the Rothschild collection of fleas (Siphonaptera) in the British Museum (Natural History). Vol. I: Tungidae and Pulicidae. British Museum, 1953.

[R38] Iyengar R. The Siphonaptera of the Indian subregion in Oriental Insects, Suppl. N° 3. Association for the study of oriental insects, University of Delhi, India, 1973.

[R39] Jarocki J.P. Zoology or general description of animals in accordance with the latest system (en polonais), Warszawa, 1838, 6, 50–52 (*in*: Rothschild N.C. *Ectoparasites*, 1921, *1*, 129-130).

[R40] Jeanselme E. & Rist E. Précis de pathologie exotique. Masson & Cie, Paris, 1909 (Puce chique, chap. 47, 641–643)..

[R41] Jeffreys M.D.W. *Pulex penetrans*: the Jigger’s arrival and spread in Africa. South African Journal of Science, 1952, 48, 249–255.

[R42] Jordan K. Two new fleas from South America (Siphonaptera). Novitates Zoologicae, 1937, 40, 307–310.

[R43] Jordan K. Suctoria, *in*: Insects of medical importance. Smart J., 2nd ed., Jarrold & Sons, 1948, 211–215.

[R44] Jordan K. Notes on *Tunga caecigena* (Siphonaptera: Tungidae). Bulletin of the British Museum (Natural History), Entomology, 1962, 12, 353–364 (avec notes et commentaires de Smit: cf. Smit F.G.A.M., 1962b)

[R45] Jordan K. & Rothschild N.C. Katalog der Siphonaptera des Königlichen Zoologischen Museums in Berlin. Novitates Zoologicae, 18, 1911, 57–89.

[R46] Jordan K. & Rothschild N.C. A new species of Sarcopsyllidae. Ectoparasites, 1921, 1, 131–132.

[R47] Karsten H. XXXIII. Contribution towards the knowledge of the *Rhynchoprion penetrans*. Annals and Magazine of Natural History, 1865, 15, 293–312.

[R48] Lewis R.E. Resume of the Siphonaptera (Insecta) of the World. Journal of Medical Entomology, 1998, 35, 377–389. 9701915 10.1093/jmedent/35.4.377

[R49] Lewis R.E. Siphonaptera. Part I – Supraspecific classification. Part II – Alphabetical genus and species list. Part III – Alphabetical species/subspecies list. Lewis R.E. (ed.), 16th ed, 1 11, 2009.

[R50] Li K.C. & Chin T.H. *Tunga callida* sp. nov., a new species of sand-flea from Yunnan (en chinois). Acta Entomologica Sinica, 1957, 7, 113–120.

[R51] Linardi P.M. & Guimarães L.R. Sifonápteros do Brasil. Museu de Zoologia – USP, FAPESP, São Paulo, Brasil, 2000.

[R52] Linné C. Von. Systema naturae, sive regna tria naturae systematice proposita per classes, ordines, genera et species. 1758-1793. Gmelin J.F., Lipsiae, 1793.

[R53] Liu Z. *et al.* Fauna sinica Insecta Siphonaptera (en chinois, résumé anglais). Science Press, Beijing, China, 1986.

[R54] Lumaret R. Faune de Madagascar, XV Insectes siphonaptères., Institut de recherche scientifique, Tananarive-Tsimbazaza, 1962.

[R55] Macchiavello A. Sifonaptera de la costa sur-occidental de America (Primera lista y distribución zoo-geografica). Boletín de la Oficina Sanitaria Panamericana, 1948, 27, 412–460.

[R56] Marcgraf G. Historia rerum naturalium Brasiliae. Amsterdam, 1648.

[R57] Pampiglione S. & Fioraventi M.L. A report of *Tunga penetrans* (Insecta: Siphonaptera) in man in Brazil in 1557. Parassitologia, 2002, 44 (Suppl. 1), 128.

[R58] Pampiglione S., Trentini M., Mattei Gentili F., Mendes J.L.X., Pampiglione C. & Rivasi F. *Tunga penetrans* (Insecta: Siphonaptera) in pigs in São Tomé (Equatorial Africa): epidemiological, clinical, morphological and histopathological aspects, Revue d’Élevage et de Médecine Vétérinaire des Pays Tropicaux, 1998, 51, 201–205.

[R59] Pampiglione S., Trentini M., Fioraventi M.L., Onore G. & Rivasi F. Additional description of a new species of *Tunga* (Siphonaptera) from Ecuador. Parasite, 2003, 10, 9–15. 12669344 10.1051/parasite/2003101p9

[R60] Pampiglione S., Trentini M., Fioraventi M.L. & Gustinelli A. Differential diagnosis between *Tunga penetrans* (L., 1758) and *T. trimamillata* Pampiglione *et al.*, 2002, the two species of the genus *Tunga* parasitic in man. Parasite, 2004, 11, 51–57. 15071827 10.1051/parasite/200411151

[R61] Patterson.B. Mammalian phylogeny *in*: Premier symposium sur la spécificité parasitaire des parasites de vertébrés. Institut de Zoologie, Université de Neuchatel, 1957, 15–49.

[R62] Pinto C. & Dreyfus A. *Tunga travassosi* n. sp. parasita de *Tatusia novemcinctus* do Brasil Boletím Biológico, São Paulo, 1927, 9, 174–178.

[R63] Pison W. De Indiae utriusque re naturali et medica. Amsterdam, 1658, 14, 289.

[R64] Ribeiro H. Sifonápteros de Angola (Insecta, Siphonaptera). Estudo sistemático e dados bioecológicos interessando à epidemiologia da peste. Instituto de Higiene e Medicina tropical, Lisboa, 1974. 4462386

[R65] Roesel Von Rosenhof A.J. Der monatlich herausgegeben Insekten-Belustigungen. II. Nürnberg, 1794.

[R66] Rothschild N.C. The generic name of the sand-flea. Ectoparasites, 1921, 1, 129–130.

[R67] Roubaud E. Une nouvelle espèce de puce-chique pénétrante parasite des rats en Chine : *Dermatophilus lagrangei* n. sp., Bulletin de la Société de Pathologie exotique. 1925, 18, 399–405.

[R68] Sakaguti K. & Jameson E.W. JR. The Siphonaptera of Japan in Pacific Insects, Monograph 3. Entomology Department, Bernice P. Bishop Museum, Honolulu, Hawaii, USA, 1962.

[R69] Segerman J. Siphonaptera of Southern Africa, Handbook for the identification of Fleas. The South African Institute for Medical Research, Johannesburg, 1994, 57.

[R70] Smit F.G.A.M. A new sand-flea from Ecuador. The Entomologist, 1962a, 95, 89–93.

[R71] Smit F.G.A.M. & Jordan K. Notes on *Tunga caecigena* (Siphonaptera: Tungidae). Bulletin of the British Museum (Natural History), Entomology, 1962b, 12, 353–364.

[R72] Smit F.G.A.M. *Neotunga euloidea* gen. n. sp. (Siphonaptera: Pulicidae). Bulletin of the British Museum (Natural History) Entomology, 1962c, 12, 365–377.

[R73] Smit F.G.A.M. Siphonaptera taken from formalin-traps in Chile. Zoologisches Anzeiger, 1968, 180, 220–228.

[R74] Smit F.G.A.M. On some adaptative structures in Siphonaptera. Folia Parasitologica (Praha), 1972, 19, 5–17. 4670804

[R75] Staden H. Wahraffitige Historia und Beschreibung einer Lantschafft der Wilden, Nacketen, Grimmingen, Menschfresser Leuthen in der Newen Welt America gelegen Marburg, 1557.

[R76] Traub R. Siphonaptera from central America and Mexico. A morphological study of the aedeagus with description of new genera and species. Fieldiana Zoology, Memoirs, 1950, 1, 1–127.

[R77] Vizy Note sur la chique au Mexique et sur son action sur l’homme. Recueil de mémoires de médecine, de chirurgie et de pharmacie militaires, 3ème série, 1863, tome 10, 306.

[R78] Wagner J. *Tunga bondari* eine neue Art der Sandflöhe. Novitates Zoologicae, 1932, 38, 248–249.

[R79] Wang D.C. The Chinese *Tunga* (Siphonaptera: Tungidae) (en chinois, titre traduit en anglais). Acta entomologica sinica, 1976, 19, 117–118.

[R80] Whiting M.F., Whiting A.S., Hastriter M.W. & Dittmar K. A molecular phylogeny of fleas (Insecta: Siphonaptera): origins and host associations. Cladistics, 2008, 24, 1–31.

[R81] Wilson D.E.& Reeder D.A.M. Mammal species of the World, 2nd edition Smithsonian Institution Press, Washington and London, 1993.

